# Accessibility of Rural Life Space on the Jianghan Plain, China: The Role of Livelihood

**DOI:** 10.3390/ijerph18031301

**Published:** 2021-02-01

**Authors:** Rongrong Zhuo, Mark Rosenberg, Bin Yu, Xinwei Guo, Mingjie Wang

**Affiliations:** 1 Hubei Key Laboratory for Geographical Process Analysis and Simulation, Central China Normal University, Wuhan 430079, Hubei, China; zhuorongrong@mail.ccnu.edu.cn (R.Z.); guoxinwei@mails.ccnu.edu.cn (X.G.); 2 College of Urban and Environmental Sciences, Central China Normal University, Wuhan 430079, Hubei, China; wangmingjie414@163.com; 3 Department of Geography and Planning, Queen’s University, Kingston, ON K7L 3N6, Canada; mark.rosenberg@queensu.ca; 4 School of Public Administration, Shandong Agricultural University, Tai’an 271000, Shandong, China

**Keywords:** rural life space, livelihood capital, livelihood transitions, multiple mediation analysis, rural China, Jianghan Plain

## Abstract

This article aims to contribute to the relationship between accessibility of rural life space and rural livelihood capital and transitions in rural central China. Employing data produced from a household survey, we developed a composite index for accessibility of rural life space incorporating spatial and temporal attributes of a household’s daily activities and then explored the mediation effect of rural livelihood capital and transitions on accessibility. Results revealed a pattern of diversification in terms of life space accessibility undertaken for daily activities across households. Both livelihood capital and transitions had significant mediation effects on the relationship between socio-economic characteristics of rural households and accessibility of rural life space. The effects of livelihood capital on livelihood transitions also influenced the path on rural households’ accessibility of rural life space. One of the implications of this article is to link rural transformation to the context of urbanization and rural access issues from a perspective of daily activity, and then to figure out the best method for rural development policy and service planning.

## 1. Introduction

What urbanization brings to resident’s economic, social and personal life and how it works have been interests of numerous studies over the years [1,2,3,4]. However, rarely have studies explored the mechanism of this process from a perspective of rural life space, especially at a micro level [5,6]. As a major part of human settlement, providing good accessibility to services and facilities for residents is in essence an important role of life space. This goal is even more urgent in rural areas where services and facilities are significantly less accessible than they are for their urban counterparts [7,8,9,10,11,12]. Regarding accessibility as a supply-and-demand system [13], while there is increasing focus on the quantity of facilities and road network structure that influence accessibility from the supply side [14,15], far less attention has been devoted to the subjective capacity that represents the demand perspective and influences accessibility. Constructing a more comprehensive framework for the measurement of accessibility of rural life space needs integration with activity-based accessibility.

In the context of globalization, urbanization and industrialization, recent decades have witnessed radical socio-economic transformation in rural China [16,17]. Life practices of rural residents have changed as rural space has undergone tremendous economic, social and environmental changes. Although the size of the urban population in China exceeded the size of the rural population in 2012, China’s rural population still remained 40.42% of the total population, or 564 million people at the end of 2018. Improving the quality of rural residents’ lives will continue to be a challenging issue confronting governments for the foreseeable future. It is complicated to improve the quality of life in rural areas because of the aging of the population and poor basic facilities [18]. One way to address this issue, is to improve accessibility to facilities and services.

How and to what extent does urbanization affect rural life space? Rural household livelihoods can provide a new perspective to explore the relationship between exogenous driving factors and local development, and people’s lives [19,20,21,22]. There is ample evidence suggesting that regional development plays an important role in rural resident’s lives through livelihood transitions [23,24,25,26,27]. Since life space represents the daily activity space in which people conduct the necessities of life, better accessibility to facilities and services in a life space undoubtedly improves the quality of life [28]. In other words, limited accessibility to life space can be a challenge to the quality of life of rural households.

Despite the increased interest in livelihood transitions and accessibility to rural life space, there has been little research that characterizes their relationships, especially in the context of rural China. Moreover, there is little survey-based work on accessibility of life spaces. Much of the rural geography research has tended to focus on changes in the physical and social environment without showing the effects on rural resident’s daily life. Taking rural central China as a study case, the aim of this article is to reveal the consequences of changes to livelihoods on the accessibility of rural life space through an examination of everyday life. We argue that the evidence from rural China would be helpful for examining the real rural life and the impact of livelihoods on it in the rest of the world.

This paper is structured as follows. Section 2 reviews the relevant literature. Section 3 proposes the hypotheses, and introduces the measurement methods, data, and study area. Section 4 describes the study results; Section 5 concludes the main points of this study, and Section 6 discusses the innovations and limitations of the paper.

## 2. Literature Review

### 2.1. Accessibility of Rural Life Space

There is no uniform definition for life space. It is generally accepted that life space is composed of the local territorial domain where most spatial interactions for basic human requirements take place [29]. A large volume of life space research has focused on the activity patterns and spatial constriction of the elderly [30,31,32,33]. Life space is an effective indicator that measures the spatial extent of movement of residents. Depending on the research objectives, the divisions of life spaces are usually living, work, shopping, entertainment, and other spaces where routine activities take place in daily life. Drawing from the methodology of life space, the daily activity space of rural residents one can examine whether there is a spatial expansion or spatial constriction phenomenon from an integrated perspective.

The spatial structure of rural life space and its interaction with human behaviors have been discussed and analyzed [34]. Most of the work on life spaces at an individual or a household level tends to focused on spatio-temporal activity patterns [35,36,37,38], activity location choice [39,40,41], or activity-related factors and mechanisms that influence life spaces [42,43]. An activity-based approach that integrates quantitative and qualitative methods has played an important role in life space analysis [44,45,46]. In addition to discovering spatial structures of facilities or urban spatial structures [47], research implies that everyday life space is highly intertwined with social segregation, health outcomes, and access to social facilities [48,49,50].

Many studies agree that aggregate measures of activities can contribute a deeper interpretation to rural life. While different types of activities space are increasingly central to human geography, far less attention has been devoted to aggregated life space in rural studies until the ‘mobility turn’ or the ‘new mobilities paradigm’ was introduced [51,52]. The mobility patterns of residents have been used to study the spatial organization of regional areas and how they are produced, reproduced and transformed [37]. The mobilities of rural residents can help with understanding the way in which travel shapes people’s everyday lives, and is increasingly becoming the paradigm for interpreting the interaction between rural resident’s lifestyle and rural places. In this sense, mobilities of rural residents have been seen as an answer to the changes in rurality, more specifically, in depopulation, marginality, and social exclusion in rural areas [53]. Inequality of rural residents in terms of reduced access to different forms of capital compared with their urban counterparts or other rural residents has made rural mobilities central to understanding rural life space.

Among the studies on rural inequalities, access to services and facilities in rural or remote areas has received increasing attention from rural studies. Recent research emphasizes the spatial distribution of food, education, and healthcare in rural regions, which are highly correlated with improvements in the well-being of rural residents. Although accessibility based upon external factors, such as physical distance, street network, quantity of facilities and services has long been one way to measure accessibility now individual or household activity-based research has attracted increasing attention in rural studies.

Over the years, accessibility measurements have been used in several scientific fields to show the relationship between people’s activities and the transportation system serving it. Research reveals the relationships between accessibility of workers, consumers, patients, students with the street networks and land use patterns in order to address the problems of home-work imbalance, spatial inequality of social services, and the efficient improvement of urban space [54,55,56]. By reviewing the literature, the concept of accessibility is generally separated into active and passive accessibility [57]. Respectively, related indicators proposed are from the perspective of activity-based and opportunity-based measurements. Many studies compare different methods of measuring accessibility, and researchers argue that the physical distance plays a limited role in predicting accessibility variations within a city, and that accessibility needs to be assess to include incorporation of spatial and temporal dimensions [50,58]. Other researchers suggest that accessibility refers to the ability of an individual to take part in a particular activity or set of activities [59].

Although the approaches for measuring accessibility have improved rapidly in recent years, not much survey-based studies have been done on accessibility of life space. Furthermore, for rural or remote areas where accessibility analysis is difficult to simulate using models because of the lower density of the population and technology, micro level data is essential to conduct rural geography research focusing on the changes in the physical and social environment and simultaneously showing the effects on rural resident’s daily lives.

### 2.2. Rural Livelihood

Regarding urban and rural regions as an integrated territorial system, one of the important consequences of urbanization is the transformation of rural areas in terms of social, economic, and the spatial reconstruction of production factors. Livelihood transition of rural households is a common phenomenon in the areas that are significantly affected by urbanization, as a result of the pursuit of higher incomes in non-agricultural sectors associated with working in urban regions. There are considerable empirical evidences suggesting that livelihoods perspectives can offer a unique starting point for the analysis of complex, highly dynamic rural regions, and have been central to rural development thinking [60].

The livelihood framework includes two main elements: livelihood capital and livelihood strategies transitions, representing inputs and outcomes respectively [60]. However, previous studies often focused on only one of them to reveal the rural development process. A much more limited body of work has, however, delivered important insight into the relationship between livelihoods capital and livelihoods strategies [61]. Similarly, Bebbington argued that analysis of rural livelihood strategies need to be thought of in terms of access to capital assets [62]. Furthermore, Longpichai’s study found a positive relationship between accessibility to livelihood capital and livelihood diversification [63]. In conclusion, livelihood capital has notable effect on livelihood strategies, and vice versa. For instance, Bhandari examined the extent to which various livelihood assets contribute to livelihood change of farm households in Nepal [64].

Besides the mutual effect of livelihood capital and livelihood transitions, there is practical evidence that implies that rural livelihood is affected by the household characteristics of the farms in a region and some broad-scale exogenous factors [65,66,67,68]. It has been suggested that age, education, and distance to proximate market have significant negative effects on households’ livelihood diversification activities [69,70]. Moreover, there is a need to link livelihood research to socio-economic transitions and policies, since rural livelihoods interact with complex political, economic and environmental processes [71,72,73,74].

One of the purposes of rural livelihood transitions is to attain higher income source and then a better well-being as a result. Many non-farm sectors, such as salaried jobs, and trade, are higher return sectors, and therefore attractive to rural households. The most basic factor for high return sectors, mainly salaried jobs, is related to the capacity level of the household head, which is mainly influenced by age, educational status, occupation, et al. [75]. Therefore, to understand whether and how livelihood has an impact on human daily activities, we need to consider the mutual effects among different aspects of livelihood and develop more explicit evidence.

### 2.3. Changes in Rural Life Space in the Context of Urbanization: A Livelihoods Perspective

In the context of rapid urbanization and industrialization, rural areas has seen a massive socio-economic transition in recent decades. Mechanization in the agricultural sector has resulted in production efficiencies, creating surplus labor and migration to urban areas where agricultural workers seek wage labor. For some of them, the situation is only temporary and they return before the beginning of the next farming season, while for others they become permanent non-farm urban workers. This process also results in rural diversification in terms of economic production and social-cultural life-style [76,77,78,79].

The effect of urbanization on daily life has received widespread attention from scholars in recent decades. Many scholars have devoted their energy to the identification and understanding the mechanisms of urbanization and their impacts on the quality of life in rural or remote areas, and to inform future policies for rural development by doing so [80].

A bunch of studies highlight the various correlation relationships between livelihood capital and livelihood strategies [63,81,82]. However, few studies have analyzed the correlates of rural residents’ daily activities in an urbanization context. A livelihoods framework can bridge the relationship between changes in economic/production and social/living variation, and thus bridge perspectives across regional and local development.

**Figure 1:**
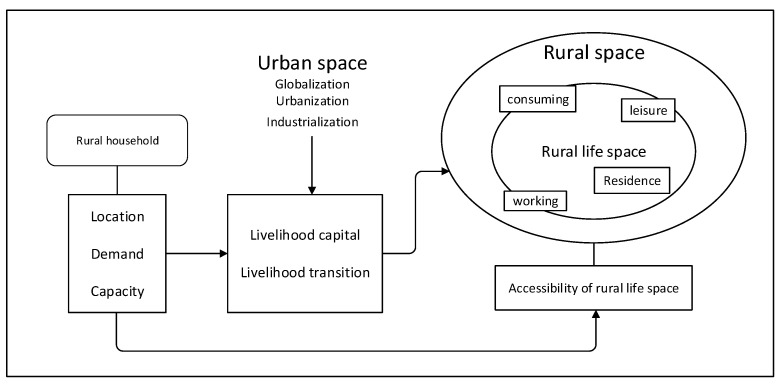
A framework for accessibility of rural life space.

In conclusion, this article intends to propose a framework for studying accessibility of rural life space (Figure 1), which can provide a practical guide to understanding what effects the spatial transformation of rural areas has on human activities and contribute to the decision-making of policy. Moreover, examining the association between accessibility to rural life spaces and rural livelihoods can provide some implications for studying changes in human activities in the context of urbanization.

The framework starts with the characteristics of rural households, including location, demand and capacity. Those characteristics directly impact on a household’s accessibility of rural life space, and also indirectly impact on it through livelihood capital and livelihood transitions. Rural life space is an important dimension of rural space and a section of the urban–rural spectrum. The processes of globalization, urbanization, and industrialization in urban space affect rural livelihood. Consequently, livelihood acts as an agency between rural households and accessibility of rural life space, and as a representative factor of urban space in rural space. The framework attempts to put rural space under the impact of urban space. It recognizes that the rural life space and the forces are complicated and dynamic. It tries to bridge the gap between rural life space and the rest of the world, which represents the gap between the micro- and macro-level.

## 3. Methods and Study Area

### 3.1. Measures

#### 3.1.1. Measure of Accessibility of Rural Life Space

The accessibility of rural life space is defined by three types of daily behavior, including consuming, leisure, and occupation behavior of rural households using the following equation:

(1) $$a_{ij}=\frac{D_{ij}F_{ij}L_{ij}}{d_{ij}}$$

(2) $$A_{i}=\sum a_{ij}$$

where *i* represents rural household, *j* represents the types of daily behavior. $a_{ij}$ represents the sub-items of accessibility of life space, $A_{i}$ represents accessibility of rural life space. $F_{ij}$ and $D_{ij}$ are frequency and duration of household i carry out daily behavior j, $L_{ij}$ is locations of daily behavior *j*. Lastly, $d_{ij}$ is the temporal distance between residence and location of daily behavior.

We assigned values for location, frequency, and duration of daily behaviors using the following questions: (1) Where/how often/how much time do you usually buy/spend at your grocery store? (2) What do you usually do in your spare time? Where/how often do you usually go to do recreation activities? How much time do you usually spend on those activities? (3) How far is your working place from your house? How often do you usually go for your work? How much time do you usually spend at your work per day? For occupation behavior, we assigned different weight to full-time farm (assigned 1), part-time farm (assigned 2) and non-farm (assigned 3) because of their different contribution to family income. The weight assigned to each category shows the difficulty levels of people accessing to a higher-paid occupation space.

The scores were calculated by multiplying the daily activities levels. The locations of life space were divided into five types, including house, village, town, county, and outside of the county, which were assigned values ranging from 1 to 5, respectively. The frequency activity performed was also classified into the following five levels: less than one time per week = 1, one time per week = 2, twice a week = 3, once every two days = 4, and daily = 5. The total score was the sum of the three types of daily behaviors.

#### 3.1.2. Measure of Rural Livelihood Capital and Transitions

Rural livelihood denotes people’s access to capital assets and the ways in which people deploy, transform, and expand those assets to make a living [83]. Given its concept, the analytical framework of rural livelihood is composed of several aspects, among which livelihood capital and livelihood transitions can provide effective help with exploring rural development and a rural household’s life.

Using the sustainable livelihoods approach [84], a rural household’s livelihoods capital can be examined by summing up human capital, natural capital, financial capital, social capital, and physical capital (Table 1). Human capital refers to the qualities and abilities of labor that influence productivity, we measured it by the number of household laborers and occupational skills of the household. Natural capital can be defined as the stocks of natural assets, it was measured by cultivated land area of the household. Social capital has been defined as “the aggregate of the actual or potential resources which are linked to possession of a durable network of more or less institutionalized relationships of mutual acquaintance and recognition” [85], it was measured by two indexes, one was a dichotomy whether any member in a household is or has been worked in public office or not, the other was neighborhood satisfaction that valued from 1 to 5. Working for public office makes people getting a larger size of network of connections easier. Financial capital is any economic resource that rural households have to finance their needs. Per capita annual income of household was considered under financial capital. Physical capital refers to the apparatus used to produce a good and services, it was measured by the number of durable consumer goods and living space per person in a household.

Rural livelihood strategy was indicated by the ratio of non-farm workers in a rural household. We divided the rural livelihood transitions into three categories: full-time farming, part-time farming, and non-farm. We defined full-time farming if the labors of a rural household are all working in agricultural sector, while non-farm refers to none of the labors of a rural household are working in agricultural sector. Part-time farming refers to the labors of a rural household are working in both agricultural and non-agricultural sector. To reveal the diversity of rural livelihood strategies from a perspective of temporal variation, we valued households by the livelihood transition in the period from 2007–2017. For that part, rural livelihood transitions were assigned values from 1~5 with a consideration of the potential income gaps between different livelihood strategies. Specifically, the 5 value-assigned categories for rural livelihood transitions were as follows: 1 = constant full-time farming, from part-time farming to full-time farming; 2 = constant part-time farming, from non-farm to part-time farming; 3 = constant non-farm, from full-time farming to part-time farming; 4 = from part-time farming to non-farm; 5 = from full-time farming to non-farm.

### 3.2. Multiple Mediation Analysis

According to the hypotheses of this study, rural livelihood capital and transitions interact with per capita annual income of household, household structure, the education background of the householder, and all of the above variables have an effect on accessibility of life space. There are also mediating effects of rural livelihood capital and transitions on the relationship between the socio-economic status of rural households and the accessibility of life space, which can be examined by conducting Structural Equation Modelling (SEM). The advantages of SEM are that it can be used to examine a sequence of relationships, e.g., *X*→*M*→*Y*, and deal with measurement error.

**Figure 2:**
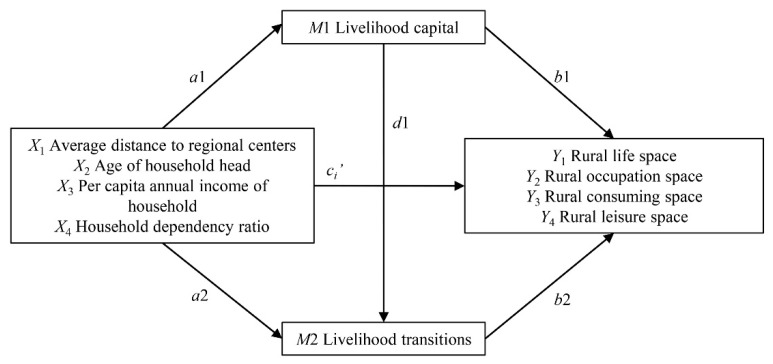
Multiple mediation model for the relation between rural households and accessibility of rural life space.

Figure 2 depicts a multiple mediation model with two mediators through which socio-economic characteristics of rural households exert their effects on the accessibility of rural life space. There are four independent variables, two mediators, and four outcomes. The sum of *X*’s direct and indirect effect on *Y*, denoted by *c*_i_ in Figure 1, is *c_i_* = *c_i_’* + *a*1 *× b*1 *+ a*2 *× b*2 + *a*1 × *d*1 × *b*2. *a*1 and *a*2 indicate the paths from *X* to the mediators (*M*1 and *M*2), *b*1 and *b*2 indicate the paths from the mediators (*M*1 and *M*2) to the outcomes (*Y*_1_ to *Y*_4_), *d*1 is the effect of *M*1 on *M*2. The direct effect of *X_i_* on *Y_i_* is given by *c_i_’*. The relative indirect effects of *X* on *Y* through *M1* and *M*2 are constructed by multiplying *a*1 and *a*2 by *b*1 and *b*2. Using the likelihood ratio (LR) test, contrasts were estimated to compare the specific indirect effects through *M*1 and *M*2, contrasts = *a*1 × *b*1 − *a*2 × *b*2. Bootstrapping was used to estimate the significance of indirect effects. Bias-corrected bootstrapping (CI), as the recommended approach, was used to detect an indirect effect [86]. Simulations were conducted using Mplus (version 7).

### 3.3. Study Area and Data Resources

**Figure 3:**
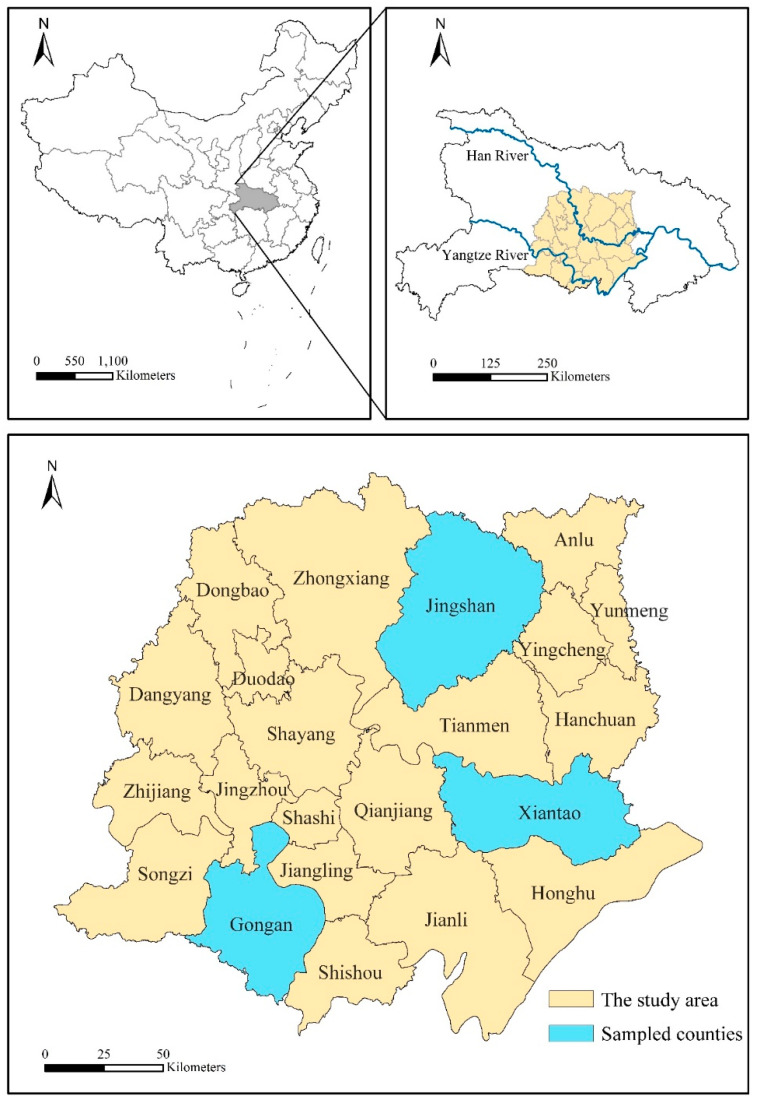
Location of the study area and sampled counties.

The Jianghan Plain is one of the main agricultural producing areas in China. It is an alluvial plain and situated in south-central Hubei Province and the middle section of the Yangtze River (Figure 3). The study area is located in the overlap between the regional development centers of Hubei Province, and the agglomeration radiation effects of these regional centers provide continuous applied force for Jianghan Plain. Households in rural villages on the Jianghan Plain are currently experiencing a radical economic and social transformation driven by urbanization and industrialization. In the period 2000–2015, the rate of urbanization of the Jianghan Plain increased from 30.53% to 52.14%, while the proportion of Gross Domestic Product (GDP) of the agricultural sector decreased from 26.31% to 13.52%. The population in the Jianghan Plain in 2015 was 1572 million, of which the rural population accounted for 47.90% (753 million).

The primary data set on which the analysis is based was collected during the months of August, September, and November 2017 in the Jianghan Plain, using a questionnaire method. 10–12 interviewers conducted the door-to-door questionnaire interviews. Each questionnaire was composed of five sections: family characteristics; housing information; spatiotemporal pattern of occupation; consuming and leisure behaviors; and living environment satisfaction. The respondents were householders or people 18 years old or over and were familiar with their families, selected randomly from the permanent population of the villages in Xiantao, Gongan, and Jingshan. Those three counties represent high, medium, and low rural development capacity levels of the Jianghan Plain, respectively [87]. We used the stratified sampling method to choose sampled households. Firstly, we divided the counties in the Jianghan Plain into three groups by assessing rural development capacity. Then, three counties, namely Xiantao, Gongan, and Jingshan, representing high-, medium-, and low-level rural development capacity respectively, were chosen. And we used the same method to choose sampled towns and villages for every county. Lastly, we randomly selected sampled households by conducting door-to-door questionnaire interviews. A total of 2446 individuals were interviewed, all of them were registered as rural residents on the *Hukou* system. All participants gave verbal informed consent. Continued participation. during the face-to-face interview was taken as evidence of continued willingness to participate. In China, ethics committee approval is not required since the survey was anonymous and the data cannot be tracked back to identifiable individuals.

## 4. Results

### 4.1. Socioeconomic Characteristics of Rural Households

Table 2 shows descriptive statistics of all the measures in 2017 by households. Most of the sampled household head were between 35–60 years old, with 35.2 percent over 60 years old, highlighting the fact that the younger generation tends to drift away from rural areas towards urban areas where there are more higher-paid employment opportunities. The number of labor in sampled households was around 2 to 4. Most families had a per capita income of about 0–10,000 Chinese yuan/year. Average distance to regional centers refers to the straight-line distance between a village and the administrative centers at the town and county level where the village is located. Euclidean distance is considered one of the most used distance metric at a larger spatial extent (e.g., county or city) [88]. Use an equal-interval classification method to split this variable value into four classes followed by reassigning a new value from 1 to 4. About 47% of the sampled households were located within 10.784–15.683 km of a regional center, while 13% of the sampled households lived more than 20 km away from a regional center.

The accessibility indices of rural life space and its subitems were also divided into four parts by applying quartering methods. As shown in Table 3, 90% of sampled households fall into the category with the lowest accessibility of rural life space, which demonstrates that there are major differences among sampled households in terms of the accessibility of rural life space. For the sub-items, 95% or above fall in the category of the lowest accessibility of rural occupation space and consumption of space. Similarly, only about 5% of sampled households have better accessibility to rural leisure space. These results imply that there are striking differences between the households in terms of accessibility of rural life space.

### 4.2. Assessments of Rural Livelihood Capital and Livelihood Transitions

After determining the household level factors of rural livelihood capital and livelihood transitions, we further estimated this index by using a weighted average method. As shown in Table 4, more than half of sampled households represent a value of livelihood capital ranging from 23 to 28. In addition, 66.231% of sampled households had a higher livelihood capital than the average. Most of the households concentrate on full-time farming, indirectly indicating that the study area is highly dependent on agricultural production. On the other hand, part-time farming and a shifting from non-farm to part-time farming make up the second largest group of livelihood strategies. At present, non-farm employment as a shift from part-time farming and full-time farming represents only 5.1 percent of all sampled households, and only a small part of the sampled households were in non-farm employment in the period from 2007 to 2017. To sum up, the rank of percentage of livelihood strategies in rural households sampled was full-time farming is most prevalent, followed by part-time farming and non-farm employment.

### 4.3. Multiple Mediation Analysis

We applied multiple mediation analysis using SEM to examine the structural relationships among age of the householder, household dependency ratio, per capita annual income of household, average distance to regional centers, and livelihood capital and livelihood transitions for accessibility of rural life space. Table 5 illustrates the results of multiple mediation analysis using 5000 bootstrap re-samples. Table 6 reported the mediation effects of independent variables on rural consuming, leisure and occupation space through livelihood capital and livelihood transitions. Significant effects were detected using bias-corrected bootstrap confidence intervals.

The results of rural life space showed that: (1) from the total effect perspective, *per capita annual income of household* had a significant positive total effect on the accessibility of rural life space. In contrast, *age of the householder* and *household dependency ratio* had a significant negative effect on the accessibility of rural life space. By comparing the total effects of independent variables on outcomes, it can be seen that the largest total effect on accessibility of rural life space came from *age of the householder*, followed by *household dependency ratio* and *per capita annual income of household*. (2) All of the independent variables had significant indirect effects on accessibility of rural life space through the effect of *livelihood capital*. The specific indirect effects of *average distance to regional centers* and *per capita annual income of household* through *livelihood capital* were significantly positive, while that of *age of the householder* and *household dependency ratio* were significantly negative. Except *per capita annual income of household*, the rest of other independent variables had significant negative indirect effects on accessibility of rural life space through the effect of *livelihood transitions.* (3) In addition, the mediation effects of *livelihood capital* and *livelihood transitions* on *accessibility of rural life space* were not consistent both in terms of effect strength and effect direction. More specifically, the specific indirect effect of *average distance to regional centers* on *accessibility of rural life space* through *livelihood capital* was significantly larger than the specific indirect effect through *livelihood transitions*, with a BC 95% CI of 0.145 to 0.384, as well as *age of the householder.* Furthermore, the specific indirect effects of *average distance to regional centers*, *per capita annual income of household* through the proposed two mediators were significantly positive, while that of *age of householder* and *household dependency ratio* were significantly negative.

The results of the multiple mediation analysis of the relation between household characteristics, livelihood and accessibility of rural consuming, leisure, and occupation space are summarized in Table 6.

(1) All of the effects *average distance to regional centers* on *accessibility of rural consuming space* were not significant. Both the total direct of *age of the householder* and *household dependency ratio* were significant and negative, and the specific indirect effect of *household dependency ratio* through *livelihood capital* was significantly lower than that of *livelihood transitions*. The total direct and indirect effects of *per capita annual income of household* were significantly positive. In addition, the specific indirect effects of *age of the householder* and *household dependency ratio* on *accessibility of rural consuming space* via the two proposed mediators were significantly negative, while *per capita annual income of household* performed in a significant positive manner.
(2) For *accessibility of rural leisure space*, the total effects of *average distance to regional centers* and *per capita annual income of household* were significant and positive. Examination of the contrasts revealed that the specific indirect effects of *per capita annual income of household* through *livelihood capital* was significantly larger than that of *livelihood transitions*, the specific indirect effects of *age of the householder* and *household dependency ratio* through *livelihood capital* were significantly smaller than that of *livelihood transitions*, whereas the specific indirect effects of *average distance to regional centers* were not significantly different from each other. In addition, the specific indirect effects of *per capita annual income of household* on *accessibility of rural leisure space* via the two proposed mediators were significantly negative, while *age of the householder* and *household dependency ratio* were significantly positive.
(3) For accessibility of rural occupation space, the total direct and indirect effects of per capita annual income of household were significantly positive, while average distance to regional centers, age of the householder and household dependency ratio were significantly negative. The examination of contrasts showed that only the specific indirect of per capita annual income of household through livelihood capital was significantly lower than that of livelihood transitions, while the other indirect effects were significantly larger. Furthermore, livelihood transitions was a significant mediator of all of the X_i_ →Y_i_ relations, while livelihood capital was not significant between average distance to regional centers, per capita annual income of household and accessibility of rural occupation space.

## 5. Discussion

Accessibility of life space is one of the determinants of quality of life. Nearly half of the world’s population now live in rural areas, their demands of life space are not different from the residents in urban areas. There are several useful approaches to describe quality of life, among which objective indictors show increasing validity [89,90]. Globally, the urbanization process is a main driving force of continuous change of rural life space, rural China is not an exception. Current studies on the effect of urbanization on rural areas focus more on changes in the rural landscape, rural-urban migration, rural development model, while the real daily life of rural households has not been considered much. To address this challenge, this study introduced rural household’s daily activity to assess the accessibility of rural life space, then estimated its mechanism from the factors that are closely connected with urbanization. By depicting rural household’s daily activity, the accessibility of rural life space offers a different interpretation of urbanization’s effect on rural.

Activity-based accessibility invites us to adjust our perspective to develop methodological tools without ignoring behavioral content. Through this new lens, accessibility to facilities and community services, or other public spaces in an urban context, could be easier estimated. More recently, for example, the emerging questions at the interface of access to public space and the COVID-19 Pandemic call for a methodology for revealing the impact of individual and environmental context on access to green areas [91,92,93]. In addition, it is worth mentioning that the measure of distance we used for calculating the rural households characteristics, which is straight-line spatial distance, is a common method used in analyzing accessibility issues but less practical [94].

Taking livelihood capital and livelihood transition as mediation factors to assess the effect of urbanization on rural life space is another contribution of this study. Rural livelihood factors have been lightly ignored and regarded merely are household socio-economic characteristics. We argue that there is a gap between urbanization and rural household’s well-being, and the livelihood perspective is one of the ways to fill. Current examinations of rural livelihood are mostly oriented to its measurement methods and change patterns, not drivers of rural life. The effect of urbanization on the accessibility of rural life space can be extended to a longer pathway providing insights of how rural household response to the urbanization process, which feeds back to knowing how policy making can meet the demand of rural household. However, there are still challenges there, the shortage of rural map data and resident’s activity data is one of them.

One of the limitations of this study is the lack of a dynamic perspective in the study. To determine whether and to what extent rural livelihood can affect the quality of life, it is helpful to explore the evolution processes of the rural life space and how they are influenced by interior and external factors. In this regard, further research should investigate both the changing pattern of rural life space and livelihood. Another issue that deserves investigation is to find what other factors within the context of urbanization have impacts on rural life, and how these factors interact and integrate to influence rural development.

## 6. Conclusions

By considering one of the main agricultural production regions of China, this paper analyzed the effects of socio-economic characteristics of rural household on the accessibility of rural life space through livelihood capital and livelihood transitions in the context of urbanization. About 90% of sampled households are in the group with the lowest accessibility of rural life space, indicating striking differences among sampled households in terms of the accessibility of rural life space. So were the sub-items. The livelihood perspective was adopted to reveal the impacts of urbanization on the study area. We found that more than half of the sampled households had higher livelihood capital than the average level and most of the households are concentrated in the livelihood strategies of full-time farming. Result indicated that the study area is still highly dependent on agricultural production, with a shifting tendency towards part-time farming and non-farm.

The mediation analysis indicated that both livelihood capital and livelihood transitions had significant mediation effects on accessibility of rural life space; however, the effects varied depending on the independent variable and different aspects of rural life space. Furthermore, all independent variables had significant indirect effects on accessibility of rural life space through the effect of livelihood capital on livelihood transitions. The specific indirect effect of average distance to regional centers and age of the householder on accessibility of rural life space through livelihood capital was significantly larger than the specific indirect effect through livelihood transitions. Surprisingly, average distance to regional centers played a least important role in accessibility of rural life space. This might apparently be explained in terms of the different accessibility requirements of different types of daily activity. In most of cases, specific indirect effects through livelihood capital were significantly larger than that of livelihood transitions.

## Figures and Tables

**Table 1:** Key variables of livelihood capital.

Components of Livelihood Capital	Proxy Indicators	Value Category
**Human capital**	C_1_ Household labor (person)	1 = 1, 2 = 2, 3 = 3, 4 = 4, 5 and above = 5
	C_2_ Occupational skills	full-time farming = 1, part-time farming = 2, non-farm = 3
**Natural capital**	C_3_ Cultivated land area (km^2^)	less than or equal to 1 = 1, 1 to 3 = 2, more than 3 to 5 = 3, more than 5 to 10 = 4, more than 10 = 5
**Social capital**	C_4_ Neighborhood satisfaction	We measured neighborhood satisfaction using a five-point Likert scale, in which “not at all satisfied”, “partly satisfied”, “satisfied”, “very satisfied”, “extremely satisfied” are used as options corresponding to the scores of 1, 2, 3, 4, and 5, respectively.
	C_5_ Whether or not work(ed) in public office	No = 0, Yes = 1
**Financial capital**	C_6_ Househould income	less than 20,000 = 1, 20,000 to 40,000 = 2, 40,001–80,000 = 3, 80,001–150,000 = 4, more than 150,000 = 5
**Physical capital**	C_7_ Number of durable consumer goods	1 = 1, 2 = 2, 3 = 3, 4 = 4, 5 and above = 5
	C_8_ Living space per person (m^2^)	less than or equal to 10 = 1, more than 10 to 20 = 2, more than 20 to 30 = 3, more than 30 to 40 = 4, more than 40 = 5

**Table 2:** Characteristics of the rural households sampled.

Variables	Assigned Values	Proportion (%)	Variables	Proportion (%)
Age of sample (years)			Household dependency ratio (%)	
under 35	1	1.4	0	22.6
35–60	2	63.4	(0, 25]	22.3
60 over	3	35.2	(25, 40]	18.6
Per capita annual income of household (Chinese yuan/year)			(40, 60]	22.1
			(60, 100]	14.4
			Average distance to regional centers (km)	
under 5000	1	47.3	[5.886, 10.784]	19.379
5000–10,000	2	32.1		
10,000–15,000	3	7.9	(10.784, 15.683]	47.097
15,000–20,000	4	7.5	(15.683, 20.581]	20.074
more than 20,000	5	5.2	(20.581, 25.479]	13.451

Notes: 1 Chinese yuan = 0.15 U.S. dollar.

**Table 3:** Accessibility of rural life space in Jianghan Plain.

Variables	Accessibility Indices	Proportion (%)	Variables	Accessibility Indices	Proportion (%)
Accessibility of rural life space	[9.5, 110.375]	89.493	Accessibility of rural consuming space	[0.2, 90.15]	98.487
	(110.375, 211.25]	10.016		(90.15, 180.1]	1.308
	(211.25, 312.125]	0.286		(180.1, 270.05]	0.123
	(312.125, 413]	0.204		(270.05, 360]	0.082
Accessibility of rural occupation space	[2, 61.5]	95.176	Accessibility of rural leisure space	[0.5, 27.875]	52.33
	(61.5, 121]	4.538		(27.875, 55.25]	42.355
	(121, 180.5]	0.245		[55.25, 82.625]	5.274
	(180.5, 240]	0.041		(82.625, 110]	0.041

**Table 4:** Classifications of rural livelihood capital and livelihood transitions.

Variables	Indices	Proportion (%)
Livelihood capital	[13, 18]	3.271
	(18, 23]	30.499
	(23, 28]	56.419
	(28, 33]	9.812
Livelihood transitions		
Constant full-time farming, from part-time farming to full-time farming, from non-farm to full-time farming	1	49.1
Constant part-time farming, from non-farm to part-time farming	2	27.5
Constant non-farm, from full-time farming to part-time farming	3	18.3
From part-time farming to non-farm	4	1.6
From full-time farming to non-farm	5	3.5

**Table 5:** Results of multiple mediation analysis of the relation between household characteristics, livelihood and accessibility of rural life space.

Variables			BC 95% CI		Variables			BC 95% CI	
		Beta	Lower	Upper			Beta	Lower	Upper
Average distance to regional centers	Total	0.252	−0.062	0.545	Per capita annual income of household	Total	4.910	3.307	6.509
	Totalind	−0.158	−0.261	−0.062		Totalind	1.775	1.271	2.416
	*a*1*b*1	0.042	0.008	0.086		*a*1*b*1	0.854	0.351	1.432
	*a*2*b*2	−0.217	−0.307	−0.133		*a*2*b*2	0.554	0.172	0.979
	*a*1*d*1*b*2	0.107	0.004	0.037		*a*1*d*1*b*2	0.367	0.231	0.549
	Contrasts	0.259	0.145	0.384		Contrasts	0.301	−0.334	0.811
Age of the householder	Total	−7.692	−10.353	−4.976	Household dependency ratio	Total	−5.299	−12.130	−1.956
	Totalind	−5.360	−6.569	−4.276		Totalind	−4.670	−11.134	−2.405
	*a*1*b*1	−1.731	−2.419	−1.097		*a*1*b*1	−2.052	−5.605	−0.829
	*a*2*b*2	−3.114	−4.150	−2.253		*a*2*b*2	−1.926	−5.016	−1.013
	*a*1*d*1*b*2	−0.515	−0.752	−0.357		*a*1*d*1*b*2	−0.692	−1.541	−0.322
	Contrasts	1.384	0.216	2.669		Contrasts	−0.125	−2.577	1.440

Notes: Beta is the standardized regression coefficient; 5000 bootstrap samples. BC 95% CI = bias-corrected 95% confidence interval. Totalind refers to total indirect effect.

**Table 6:** Results of multiple mediation analysis of the relation between household characteristics, livelihood and accessibility of rural consuming, leisure, and occupation space.

Variables	Consuming				Leisure			Occupation		
			BC 95% CI			BC 95% CI			BC 95% CI	
		Beta	Lower	Upper	Beta	Lower	Upper	Beta	Lower	Upper
Average distance to regional centers	Total	0.089	−0.234	0.405	0.345	0.216	0.489	−0.181	−0.324	−0.025
	Totalind	−0.007	−0.053	0.039	0.038	0.013	0.066	−0.189	−0.270	−0.011
	*a*1*b*1	0.019	−0.001	0.050	0.024	0.005	0.048	−0.001	−0.012	0.006
	*a*2*b*2	−0.028	−0.077	0.003	0.015	0.000	0.033	−0.204	−0.281	−0.130
	*a*1*d*1*b*2	0.002	0.000	0.010	−0.001	−0.003	0.000	0.016	0.003	0.033
	Contrasts	0.047	0.011	0.108	0.009	−0.015	0.038	0.203	0.109	0.303
Age of the householder	Total	−2.437	−4.179	−0.706	−0.362	−1.666	0.941	−4.892	−6.382	−3.614
	Totalind	−1.177	−1.738	−0.647	−0.771	−1.226	−0.409	−3.411	−4.221	−2.655
	*a*1*b*1	−0.755	−1.303	−0.238	−1.084	−1.445	−0.765	0.108	−0.204	0.417
	*a*2*b*2	−0.363	−0.973	0.129	0.268	0.047	0.493	−3.020	−3.853	−2.298
	*a*1*d*1*b*2	−0.060	−0.140	−0.003	0.044	0.009	0.087	−0.499	−0.687	−0.357
	Contrasts	−0.392	−1.025	0.258	−1.352	−1.816	−0.931	3.128	2.250	4.053
Per capita annual income of household	Total	1.577	0.619	2.546	1.390	0.765	2.123	1.943	1.004	3.018
	Totalind	0.522	0.207	0.844	0.503	0.278	0.819	0.750	0.369	1.157
	*a*1*b*1	0.411	0.104	0.758	0.595	0.336	0.887	−0.151	−0.421	0.099
	*a*2*b*2	0.067	0.009	0.181	−0.055	−0.122	−0.018	0.542	0.158	0.929
	*a*1*d*1*b*2	0.044	0.003	0.096	−0.036	−0.067	−0.011	0.359	0.228	0.506
	Contrasts	0.344	0.042	0.621	0.649	0.368	0.931	−0.692	−1.101	−0.306
Household dependency ratio	Total	−1.186	−3.443	0.256	−1.672	−2.784	0.061	−2.441	−5.150	−1.070
	Totalind	−1.252	−2.826	−0.608	−0.943	−2.273	−0.436	−2.474	−4.820	−1.478
	*a*1*b*1	−0.919	−2.081	−0.378	−1.183	−2.664	−0.612	0.051	−0.356	0.497
	*a*2*b*2	−0.245	−0.748	−0.030	0.176	0.035	0.444	−1.858	−3.867	−1.116
	*a*1*d*1*b*2	−0.088	−0.233	−0.011	0.063	0.014	0.160	−0.667	−1.291	−0.368
	Contrasts	−0.674	−1.663	−0.065	−1.359	−3.021	−0.718	1.909	0.984	4.850

Notes: Beta is the standardized regression coefficient; 5000 bootstrap samples. BC 95% CI = bias-corrected 95% confidence interval.

## Data Availability

Not applicable.
